# Establishment of immunoassay for detecting HPV16 E6 and E7 RNA

**DOI:** 10.1038/srep13686

**Published:** 2015-09-03

**Authors:** Sen Ding, Steven Y. Qian, Yang Zhang, Wenlei Wu, Gensheng Lu, Yan Lu, Xiujing Feng, Li Li, Pingping Shen

**Affiliations:** 1 State Key Laboratory of Pharmaceutical Biotechnology, Nanjing University, Nanjing, 210023, China; 2 MOE Key Laboratory of Model Animal for Disease Study, Model Animal Research Center, Nanjing Biomedical Research Institute, Nanjing University, Nanjing, 210061, China;; 3 Department of Pharmaceutical Sciences, North Dakota State University, Fargo, ND 58108-6050, United States; 4 Institute and Hospital of Stomatology, Nanjing University Medical School, Nanjing, 210093, China; 5 Nanjing Maternity and Child Health Care Hospital, Nanjing, 210004, China; 6 School of Chemistry & Life Sciences, Nanjing University Jinling College, Nanjing, 210089, China

## Abstract

Cervical carcinoma is the most prevalent malignancy second only to breast cancer among women worldwide. Since more than 99% of cervical cancers are caused by human papilloma virus (HPV), measurement of HPV (HPV test) was commonly used in screening risk and/or early stage of cervical cancer as well as assessing the efficacies of the treatments that can decrease the incidence of cervical cancer. Many approaches that diagnose HPV infections have been developed, while most of them have distinct shortcomings. We here established a novel immunoassay method in which the pairs of unlabeled DNA probes firstly bind to HPV16 E6 and E7 RNAs to form the DNA-RNA hybrids, and the hybrids will subsequently be identified by S9.6 antibody. The sensitivity of this highly specific method can reach ~0.923 pg/mL and ~0.424 pg/mL of *in vitro* transcribed HPV16 E6 and E7 RNA, respectively, and reverse transcription and polymerase chain reaction (PCR) amplification were no longer needed. Thus, our immunoassay approaches can precisely reflect the actually viral load that is related to the course of HPV infection. In addition, it has also fast and low cost characteristic feature.

Human papilloma virus (HPV), circular double-stranded oncogenous DNA virus, belongs to *Papillomaviridae*, *Alpha-Papillomavirus genera*. HPV can be transmitted through intimate contact and was firstly found in the 1970s^1^,^2^. There are more than 100 types of HPVs, and many of them have been shown to be responsible for the development of multiple epithelial malignancies, e.g., most notably cervical cancer^3^. Currently, cervical carcinoma has been the most prevalent malignancy second only to breast cancer among women worldwide. More than 99% cervical cancers are caused by HPV infection^4^. In addition, the majority of cervical cancer cases (~70%) are caused only by infection of few types of HPVs, e.g., HPV genotypes 16 and 18^5^,^6^,^7^, and the E6 and E7 genes of these HPV oncogenous viruses as well as their transcription and/or translation products that can significantly affect the ability of virus invasion^8^,^9^,^10^,^11^.

Many approaches to diagnose HPV infections have been developed^9^,^12^,^13^,^14^,^15^,^16^. To date, however, as HPVs cannot have been cultured *in vitro*, culture-based techniques are generally unfeasible^9^. There were also no appropriate serological or protein-based approaches since the immunoassay that directly detects HPVs is typically limited by insufficient specificity and sensitivity to viral proteins^8^. The methods using HPV proteins as the detecting targets won’t be able to develop to effective approaches as the false-positive results are more likely to appear. Other HPV detection assays have been also developed relying on detection of viral nucleic acids (DNA, RNA)^12^,^13^. To detect DNAs of HPV can be approximately classified into three categories: *in situ* hybridization and DNA sequencing which detect the target nucleic acids directly^13^; signal amplification methods, e.g., branched DNA assays^14^, hybrid capture system^15^, and cervista HR HPV test^13^,^17^; and target amplification assays, e.g., Real-Time PCR^13^,^15^,^16^,^18^,^19^,^20^ (especially, the Roche Cobas HPV Test which was approved by the US FDA as the first-line primary screen of cervical cancer in 2014), and detection of integrated papillomavirus sequences PCR (DIPS-PCR)^15^,^21^. There are also the methods based on RNA sequences: amplification of papillomavirus oncogene transcripts (APOT) (reverse-transcriptase PCR)^22^, nucleic acid sequence-based amplification (NASBA)^13^, and transcription mediated amplification (TMA)^13^,^15^.

However, methods aiming to detect nucleic acids (DNA, mRNA) have also obvious shortcomings, including very complicated operations and the associated high cost, need of amplification, and involvement of varies of instruments that may not able to reflect the actual viral load relevant to the patient’s course of disease as well as the risk of virus transmission^15^. For example, the method of Hybrid Capture 2 for measuring the genome DNA of HPV shows that the high risk HPV more than 1 pg/mL (100,000 HPV copies) is significantly positive in 97.5% of CIN (Cervical Intraepithelial Neoplasia) II-III, and 100% of CIN III or 100% of cervical carcinoma^23^,^24^,^25^. Therefore, there is an urgent need to develop a method which could overcome these shortcomings, typically, the methods of diagnosing HPV infections involve the nucleic acid hybridization-based assays without the amplification of target HPV nucleic acids.

In this study, we established a novel immunoassay that utilize S9.6 antibody to recognize special DNA–RNA hybridization, e.g., hybrids of high-risk HPV16 E6 and E7 RNAs with DNA probes. Using pairs of unlabeled DNA probes which can bind different positions of the HPV16 E6 and E7 RNAs, the method not only reduces the cost of modification, but also increases the sensitivity of the assay. In addition, the monoclonal antibody S9.6 which was originally generated in mice by immunization with a ΦX174 bacteriophage-derived synthetic DNA–RNA antigen^26^,^27^ was characterized with high specificity and affinity to DNA–RNA hybrids^27^,^28^,^29^. Thus, this method can precisely demonstrate the actual viral load from patient as we can directly measure the RNA translated products that significantly affect the ability of virus invasion. It is also a convenient, fast, but low cost approaches along with high sensitivity and specificity.

## Results

### Immunoassay detection of synthetic DNA-RNA hybrids

**Figure 1:**
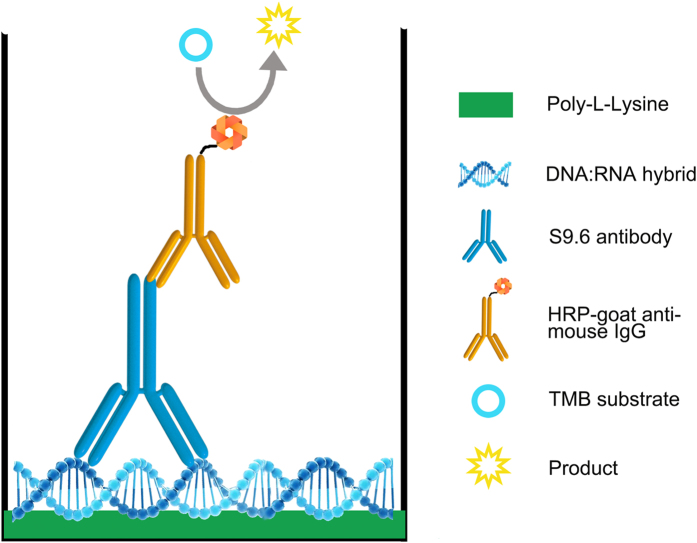
The scheme of our experimental design. The figure was drawn by Sen Ding.

Our schematic procedure of immunoassay experiment is shown in Fig. 1. The Poly-L-Lysine (PLL) at a certain concentration is firstly coated in ELISA plate. After washing by PBST buffer 3–5 times, 30 s each, 1% BSA blocking solution will be added, and DNA-RNA hybrids that are captured by PLL could thus be recognized by the S9.6 primary antibody. The HRP-goat anti-mouse IgG (H + L) that could recognize the S9.6 primary antibody will subsequently catalyze TMB substrate to a blue substance. The plate will then be read by a microplate reader following the terminate step by using stop solution to change blue substance to yellow.

**Figure 2:**
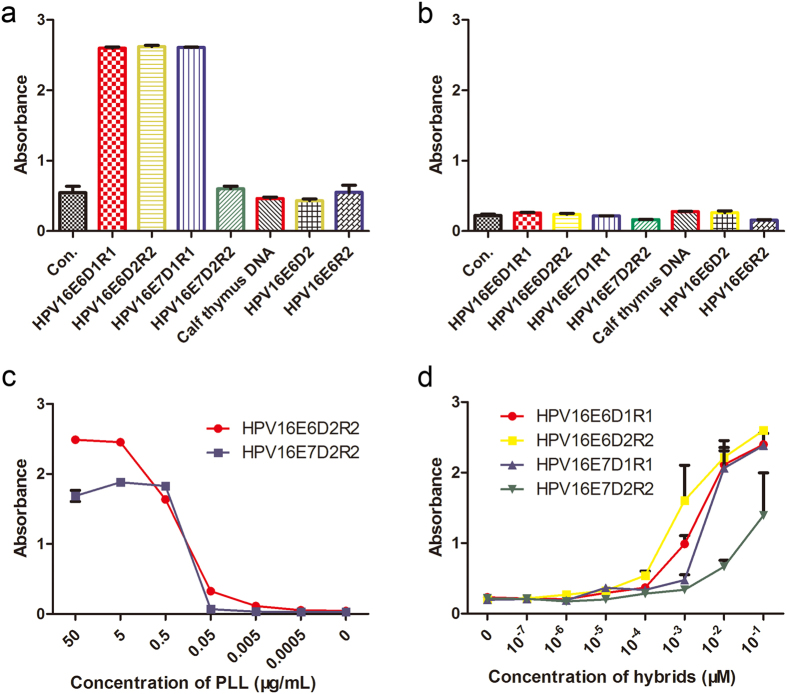
Sensitivity tests of immunoassay for synthetic DNA-RNA hybrids. DNA-RNA hybrids (HPV16E6D1R1 - 40 bp, HPV16E6D2R2 - 36 bp, HPV16E7D1R1 - 32 bp and HPV16E7D2R2 - 30 bp), double strand DNA (calf thymus DNA), single strand DNA (HPV16E6D2 - 36 nt), and RNA antigens (HPV16E6R2 - 36 nt) were detected by the immunoassay using two different pretreated microtiter plates: (**a**) by Poly-L-Lysine; (**b**) UV. Sensitivity tests of: (**c**) optimized concentration of Poly-L-Lysine for HPV16E6D2R2 (36 bp) and HPV16E7D2R2 (30 bp) hybrids, respectively, and (**d**) immunoassay for synthesis DNA and RNA probes of HPV16 E6 and E7.

Using our procedure, we first investigate the effects of pre-treatment conditions on microtiter plates and the affinity of the primary antibody with different types of antigens. When the microtiter plates were pretreated by PLL (Fig. 2a) or by UV (Fig. 2b) along with different types of nucleic acid antigens (sequences and lengths were listed in Table 1), including the DNA-RNA hybrids (e.g., HPV16E6D1R1, HPV16E6D2R2, HPV16E7D1R1 and HPV16E7D2R2), double strand DNA (e.g., calf thymus DNA), single strand DNA (e.g., HPV16E6D2), and RNA antigens (e.g., HPV16E6R2), the PLL-treated microtiter plates had very strong affinity to the DNA-RNA hybrids. However, binding with any types of nucleic acids were almost not observed in UV-treated microtiter plates, very likely because the uniformly UV-treatment for polystyrene microplates is impossible, and there is also much lower detection sensitivity of UV-treated plates vs. PLL-treated plates.

In order to optimize our experiment condition, we tested effect of PLL concentration on the signal/noise ratio (Fig. 2c). High PLL concentrations (>0.5 μg/mL) led a nonspecific combination and higher background signal, and also increased the experimental cost. We found when the PLL was coated at concentration ~0.5 μg/mL, the nucleic acid antigens could be efficiently fixed on the plate and background signal could also be minimized. In addition, this optimized PLL concentration also minimized the possible impacts of using two different lengths of nucleic acid antigens on similar and/or same detection consequence for varies of antigenic properties. When HPV16E6D2R2 of 36 bp and HPV16E7D2R2 of 30 bp hybrids (Table 1) were used to study the effect of PLL concentration on detection limit, we found that the absorbance of PLL > 0.5 μg/mL was different for varies of lengths of nucleic acid antigens to be used even the concentrations of nucleic acid antigens were the same. In contrast, once PLL concentration (<0.5 μg/mL) was used, the ability of antigens binding to PLL was significantly decreased. These data indicated that the optimal PLL concentration is 0.5 μg/mL.

We also found that different lengths of hybrid fragments had the impact on their affinities to antibody (Fig. 2c). We further compared the affinity of the primary antibody to different lengths of DNA-RNA hybrids with the same procedure. When the length of hybrid fragment was <30 bp, the binding ability of antibody to hybrid antigen was very low, and the affinity was also affected by the hybrid antigen in a strong concentration-dependent manner (Fig. 2d). When the length of hybrid antigens is >30 bp, however, the antigens could strongly bind to primary antibody and be detected as low as 10^−10^ M. The sensitivities of the procedure to detect different lengths of synthetic DNA-RNA hybrids were also studies (Fig. 2d) and DNA probes (longer than 30 nt) were found to be able to enhance detection sensitivity.

### Clonal analysis of gene template and Immunoassay of HPV16 E6 and E7 RNA

**Figure 3:**
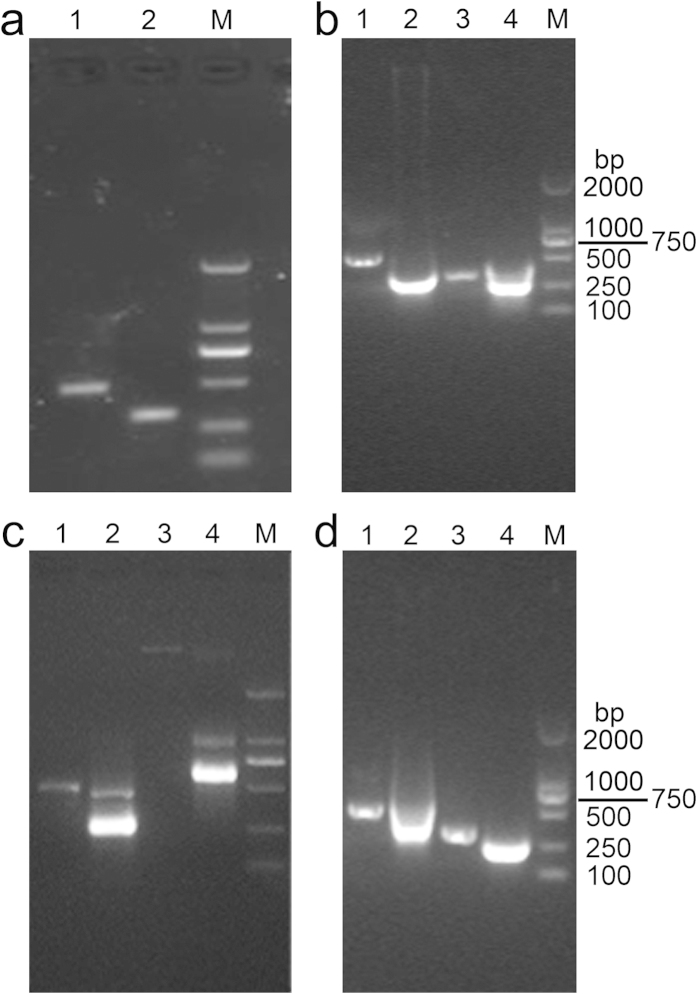
Agarose gel electrophoresis of HPV16 E6/E7 DNA and RNA. (**a**) The PCR products of HPV16 E6 and HPV16 E7 (lanes 1 and 2); (**b**) The PCR products of HPV16 E6 and their *in vitro* transcription products without DNase I treatment (lanes 1 and 2), and the PCR products of HPV16 E7 and their *in vitro* transcription products without DNase I treatment (lanes 3 and 4); (**c**) 2% agarose gel electrophoresis of PCR products of HPV16 E6 and their *in vitro* transcription products without DNase I treatment (lanes 1 and 2), 2% agarose gel electrophoresis of quality control template and its *in vitro* transcription products without DNase I treatment (lanes 3 and 4); and (**d**) The PCR products of HPV16 E6 and their *in vitro* transcription products with DNase I treatment (lanes 1 and 2), and the PCR products of HPV16 E7 and their *in vitro* transcription products with DNase I treatment (lanes 3 and 4). M: DL2000 DNA Marker.

In order to test whether our procedure can also be used to larger RNA fragment, we amplified E6/E7 from HPV16+ cervical cancer cell genomic DNA, and then cloned it into a vector containing a T7 promoter that enables the downstream *in vitro* transcription. The PCR products were checked by the agarose gel electrophoresis (Fig. 3a) and their lengths were confirmed the same as the standard sequences. We then constructed HPV16 E6 and E7 genes into pMD-19T plasmid carrier. The positive recombinant plasmids had been sequenced and analyzed by blast in NCBI GenBank to confirm the sequences. We found HPV16 E6 and E7 genes were constructed into pMD-19T and with the T7 RNA polyase promoter. The HPV16 E6 and E7 RNAs were transcribed *in vitro* as the instruction by using HPV16 E6 and E7 gene fragments as templates, and were then amplified from two recombinant plasmids by PCR. The PCR products of recombinant plasmids and RNAs transcribed *in vitro* were checked by agarose gel electrophoresis (Fig. 3b).

To investigate whether the immunoassay method could be applied to detect HPV16 E6 and E7 RNAs produced by the protocol above, we applied the synthetic probes (Table 2) to detect the RNAs. However, besides the hybrids of DNA probes along with the target RNA, the target RNA without the addition of any probes (negative control) also showed a strong signal (data not shown). It could be the result of the hybridization of the remaining DNA templates and RNA in the system. The RNAs products which were transcribed *in vitro* and contained small amount of DNA templates were thus confirmed by 2% agarose gel electrophoresis (Fig. 3c). We observed a weak light stripe of DNA above the strong light stripe of RNA (lanes 2 and 4). In order to avoid residual DNA templates from interference of the results, we pretreated the products with DNase I during the process of the RNAs preparation (Fig. 3d), and thus only RNA stripes could be observed (lanes 2 and 4). The viral products we used for interference test later had also been treated with DNase I.

**Figure 4:**
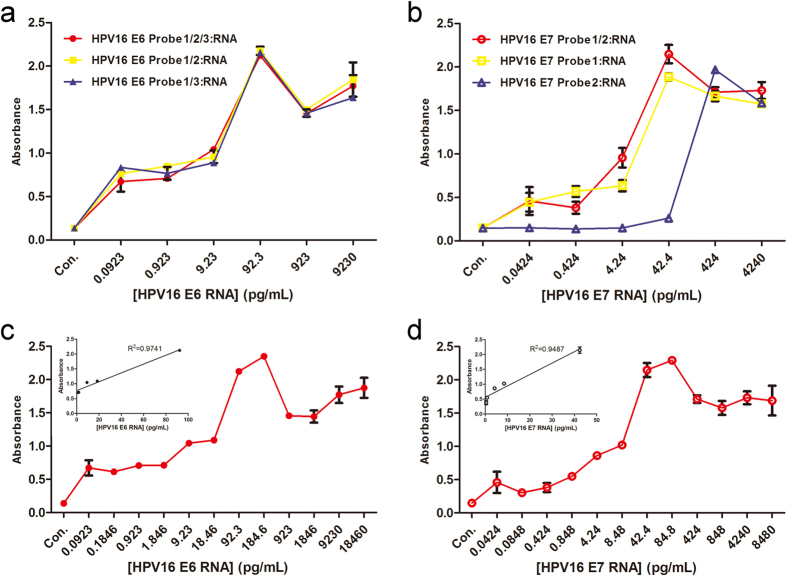
Sensitivity studies of immunoassay for HPV16 E6 and E7 RNA. (**a**) The hybridization of HPV 16 E6 probe 1 to 3 mixture (red), HPV16 E6 DNA probe 1 and 2 mixture (yellow) and HPV16 E6 DNA probe 1 and 3 mixture (blue) with gradient dilution of *in vitro* transcription products of HPV16 E6; (**b**) The hybridization of HPV16 E7 DNA probe1 and 2 mixture (red), HPV16 E7 DNA probe 1 (yellow) and HPV16 E7 DNA probe 2 (blue) with gradient dilution of HPV16 E7 RNA; (**c**) The hybridization of HPV 16 E6 probe 1 to 3 mixture with gradient dilution of HPV16 E6 RNA and the linear relationship was shown in the insertion; and (**d**) The hybridization of HPV 16 E6 probe 1 to 3 mixture with gradient dilution of HPV16 E7 RNA and the linear relationship was shown in the insertion.

In order to investigate whether the different combination of probes could affect the detection sensitivity, we applied different combination of probes to detect RNAs which were managed by gradient dilution (Fig. 4a–b). The probes with their different lengths could bind different positions on the target RNA. For example, three mixtures of probes, e.g., E6 DNA probe 1 to 3 mixture, E6 DNA probe 1 and 2 mixture, and E6 DNA probe 1 and 3 mixture, were hybridized with gradient dilution of E6 RNA (Fig. 4a), respectively. In addition, E7 DNA probe 1 and 2, E7 DNA probe 1, and E7 DNA probe 2, were also hybridized with gradient dilution of E7 RNA, respectively (Fig. 4b). We found that both the number and the length of the probes could affect the detection sensitivity in a linear range. The optimized combination of probes for the detection are: the E6 RNA with E6 DNA probe 1 to 3 mixture, and E7 RNA with E7 DNA probe 1 and 2 mixture, respectively. The immunoassay for hybridization of HPV 16 E6 probe 1 to 3 and HPV 16 E6 RNAs was administrated by a gradient dilution (Fig. 4c), while immunoassay for hybridization of HPV 16 E7 probe 1 and 2 and HPV 16 E7 RNAs was managed by a different gradient dilution as shown in Fig. 4d. The results indicated that the HPV16 E6 RNA transcribed *in vitro* could be detected by the mixture of probes at the minimum concentration of 0.923 pg/mL along with a linear relationship (R^2 ^= 0.9741 between 92.3 pg/mL and 0.923 pg/mL, inset of Fig. 4c). On the other hand, we could detect HPV 16 E7 RNA at the minimum concentration of 0.424 pg/mL along with linear concentrate range from 42.4 pg/mL to 0.424 pg/mL (R^2 ^= 0.9487, inset of Fig. 4d).

### Specificity Study of the immunoassay

**Figure 5:**
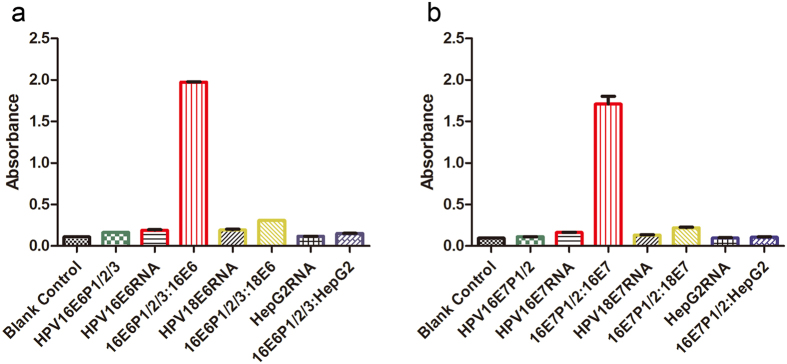
Specificity studies of immunoassay. (**a**) The hybridization of HPV 16 E6 probe 1, 2 and 3 mixture (10 μM) with *in vitro* transcription products of HPV16 E6 (92.3 pg/mL), HPV18 E6 (119 pg/mL) and HepG2 (26.9 pg/mL) total RNA; and (**b**) The hybridization of HPV 16 E7 probe 1 and 2 mixture with *in vitro* transcription products of HPV16 E7 (42.4 pg/mL), HPV18 E7 (62.8  pg/mL) and HepG2 (26.9 pg/mL) total RNA.

In order to confrim our optimized combination probes, e.g., 16 E6 DNA probes 1 to 3 mixture and 16 E7 DNA probes 1 and 2, won’t be hybridized with other vial products, we applied the HPV 18 vrial products, including 18 E6 and E7 RNA, and HepG2 total RNA in our system to test whether there are non-specific bindings of the DNA probes (Fig. 5). According to the linear responses in these sensitive experiemnts (Fig. 4c–d), we conducted specific study of 10 μM 16 E6 DNA probes 1 to 3 with HPV16 E6 RNA, HPV18 E6 RNA and HepG2 total RNA at concentrations range from 0.923 to 184.6 pg/mL, and specific study of 10 μM 16 E7 DNA probes 1 and 2 with HPV16 E7 RNA, HPV18 E7 RNA and HepG2 total RNA at reange from 0.424 to 84.8 pg/mL. Only 16 E6 RNA and 16 E7 RNA bond with the related DNA probe were observed (absorbance 2.0 and 1.75 at wavelenght of 450 nm, resepctively), while the absorbances of experiments of 18 E6 and E7 RNA, and HepG2 total RNA along with both DNA probes showed no diffenrent than the relevnt controls, including blank controls (Fig. 5a–b). Our data domonstrated that there were no non-specific bindings between the other vial products and the unlabeled DNA probes (e.g., our optomized combination probes), and our method is highly specific method.

### Immunoassay for detecting HPV+ cell lines and clinic samples

**Figure 6:**
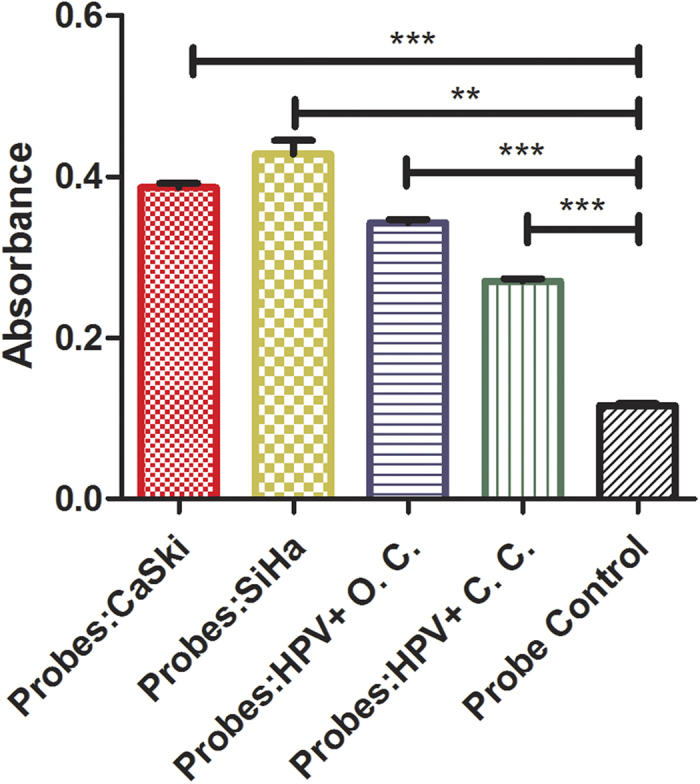
Immunoassay of HPV+ cell lines and clinic samples. The CaSki and SiHa cell lines were selected as they are HPV16+ cell lines. HPV16+ O. C. represented the hybridization of a total of RNA in HPV16+ oropharyngeal carcinoma cells along with the mixture of HPV16 E6 and E7 DNA probes, while HPV16+ C. C. represented the hybridization of the total of RNA in HPV16+ cervical cancer cells along with the mixture of HPV16 E6 and E7 DNA probes. The total RNA of all samples ~0.5 μg/mL were used in the test. The mixture of HPV16 E6 and E7 DNA probes (10 μM) was used for controls.

The CaSki and SiHa cell lines and tissues of patients with HPV16+ cervical cancer and oropharyngeal carcinoma were also tested for the assay’s clinical application. CaSki and SiHa cell lines are both HPV16+; although having slightly different numbers of DNA copies, the mRNA level in SiHA was reported higher than that in the CaSki cells^30^. After the total of RNA in these smaples were extracted, and the HPV tests were then conducted for their hybridization with the mixture of HPV16 E6 and E7 DNA probes. Significantly greater absorbances for CaSki and SiHa cell lines (vs. probe control) and much greater absorbance in SiHA cell line (vs. CaSki) were observed. The results of immunoassay of HPV16+ clinic samples indicated that our assay could be used to detect HPV in CaSki and SiHa cell line (HPV16+), tissue of HPV16+ oropharyngeal carcinoma and HPV16+ cervical cancer. The mixture of HPV16 E6 and E7 DNA probes can be used to capture the targets of RNA in relatively complicated clinical samples. As the total RNA concentration in each sample was normallized ~0.5 μg/mL in Fig. 6, the intensty of absorbance might actully represent the amount of virus in the tested cells or tiuss samples. For example, dection of the greater absorbance in SiHa cell line (vs. CaSki) indicated that the higher mRNA level in SiHa comparing with that in CaSki. Thus, our assay might allow us to conduct virus quantification for patients’ samples to provide information between the amount of virus *in vivo* and pathogenic potential.

## Discussion

Hybrid Capture 2 (HC2, Digene and now marketed by Qiagen) has been a US-FDA approved method for the routine detection of hr-HPV infection since 2003^13^. Unlike many other nucleic acid-based detection methods, HC2 and our immunoassay methods do not involve any sample amplification, and both have relatively higher sensitivity and selectivity. However, as requiring the capture antibody that is produced from the antiserum and the 8kb RNA probe hybrided with the virus DNA, HC2 method has estimated ~$40–60 per test^31^,^32^. In addition, actual number of the live viruses cannot be revealed in HC2 method^33^. We here developed the immunoassay which not only significantly decreases the cost (~$1 per test), but only owns the advantage of revealing actual number of the live viruses from the patients for the first time.

In order to decrease the cost of our immunoassay procedure, the unlabeled pairs of DNA probes were used, and microplates were also pretreated by Poly-L-Lysine. Unlike DNA or RNA probes, mostly biotin modification^34^,^35^,^36^,^37^,^38^,^39^ or radiolabeling^26^,^27^,^40^,^41^, were one-to-one hybrided with the RNA target in other studies (e.g., involve more expensive probe synthesis, and have too low sensitivity to fit the trace measurement), we used pairs of unlabeled oligonucleotides (probes) to bind the different positions of long chain in target RNA. Since unlabeled oligonucleotides were relatively simple, stable and easy to store, our immunoassay not only decreased cost, but also significantly improved method sensitivity by increasing the recognition position of antibody from many formed fragments of hybridization.

Unlike in HC2 in which microtiter plates was antiserum preincubated, the microtiter plates in our method were pretreated the by Poly-L-Lysine, a firmer, simpler and lower cost procedure; in addition, no longer need to concern about antibodies orientation problem. Certain doses of UV-treated polystyrene microplates were previously used by Zouali M and Stollar BD for measurement of antibodies to nucleic acid antigens. By comparing the method of UV-treated (via 120 μW UV germicidal lamp with distance of 65 cm 15 min to 15 h) with plates pre-coated with 50 μg/mL poly-L-lysine^42^, they found that the use of UV-irradiated plates to immobilize nucleic acid antigens provides a simple, rapid, and specific ELISA for measuring anti-nucleic acid antibodies. However, UV-treated polystyrene microplates could not bind with the nucleic acids effectively (Fig. 2b), perhaps due to the polystyrene microplates couldn’t be treated uniformly by the UV in our experiment, and there is also much lower detection sensitivity of UV-treated plates vs. PLL-treated plates. In addition, the concentration of poly-L-lysine introduced by Aotsuka S *et al*., for quantitating anti-double-stranded deoxyribonucleic acid antibodies (anti-dsDNA) experiment was 40 μg/mL^41^, while the concentration more than 0.5 μg/mL in our study already has already background signal (Fig. 2a–c).

Based on the linear responses shown in Fig. 4, we selected six concentration gradients but different range for tested RNAs (e.g., E6 range from 0.923 to 184.6 pg/mL, while E7 range from 0.424 to 84.8 pg/mL) to conduct our specific study. The result of one concentration from each tested RNA was shown in Fig. 5, e.g., absorbance of experiments of HPV16 E6 DNA probes 1 to 3 mixture with HPV16 E6 RNA (92.3 pg/mL), HPV18 E6 RNA (119 pg/mL) and HepG2 total RNA (26.9 pg/mL), respectively in Fig. 5a; absorbance of experiments of HPV16 E7 DNA probes 1 and 2 with HPV16 E7 RNA (42.4 pg/mL), HPV18 E7 RNA (62.8 pg/mL) and HepG2 total RNA (26.9 pg/mL), respectively in Fig. 5b. However, for all tested range of RNA concentration of all samples, our probes only hybrided with the target RNA and thus have very high specificity.

For the very first time, without any types of amplifications, our immunoassay can reveal the actual number of the live viruses. It is previously impossible for all other existing methods. DNA is the all target of commercial kits for HPV screening such as HC2 and the Roche Cobas HPV Test. In CH2, although the 8kb RNA probes that is used to detect the virus DNA with no need of amplication, the amplifying the target DNA templates was essential. Both tests could not reveal the actual number of the live viruses because the dead viruses might be miss-counted in their DNA samples, while only the number of the live viruses could not actually reflect the process of the infection. However, the corresponding viral load could be detected more reliably in our immunoassay as the result of the detection target is the transcribed RNA. In addition, as our targets are the transcribed RNA fragments, no requirement of reverse transcription or amplification. Thus, our method can be used for clinical diagnosis as it can provide relationship between the amount of virus *in vivo* and its pathogenic potential.

Although having good sensitivities from tested cell lines and clinical samples (Fig. 6), the RNA absorbance intensities were little too low (<1.0). The main reason could be that the probes in our current assay were only designed for HPV 16 E6 and E7 RNA. Our on-going research focuses on complementing the current probes (e.g., HPV16 E6 and E7) with the probes of other HPV16 gene fragments, such as E1/E2/E5/L1/L2 to increase signal intensity. In addition, we are also on the way to design other types of HPVs’ probes, such as HPV 18, in order to further optimize HPV assay as a high throughput screening along with virus quantification feature.

In summary, we established a sensitive, specific, rapid and low cost immunoassay that easy to be widely applied for detecting HPV16 E6 and E7 RNA without labeling. For the future, its may have the great potential to be used as a primary screen of cervical cancer in clinic.

## Methods

### Preparation of samples of synthetic DNA-RNA hybrids

Four couples of DNA probes and their complementary RNA probes were designed according to NCBI published HPV16 E6 and E7 gene sequence (NCBI Reference Sequence: NC_001526.2) and synthesized by Invitrogen Corporation. The sequences of probes were listed in Table 1. DNA and RNA probes were then hybridized as following: 10 μl of 10×annealing buffer solution (100 mM Tris-HCl, pH 7.5, 10 mM EDTA, 1 M NaCl), 1 μl of 10 μM DNA probe and 1 μl of 10 μM RNA probe (both probes complementary together), and 88 μl of DEPC H_2_O. The four kinds of mixtures were heated to denature at 95 °C for 5 min, and then cooled to ambient temperature. The same concentration of calf thymus DNA, HPV16 E6 DNA and RNA probes were treated, respectively, with above annealing system as control.

### Immunoassay for detecting synthetic DNA-RNA hybrids

Wells of polystyrene plates (Costar) were pretreated by two different ways: coated with 150 μl gradient diluted poly-L-lysine (molecular weight: 7000–15000) solution at room temperature for 30 min or overnight at 4 °C, and irradiated by UV^42^ overnight. The pretreated plates were washed 5 times by PBST (NaCl 137 mM, KCl 2.7 mM, Na_2_HPO_4_ 10 mM, KH_2_PO_4_ 2 mM, pH7.4, 1% Tween-20) and then 200 μl 1% bovine serum albumin in PBST buffer was added for 2 h incubation to block residual protein-binding sites. The plates were then washed by PBST 5 time as well, and then coated with 100 μl DNA-RNA hybrid antigens for 1 h. Wells were washed again, and 100 μl of S9.6 primary antibody (S9.6 monoclonal antibody cell line was purchased from ATCC, diluted to 1 in 500 with PBST) was added and incubated for 1 h. After washing again, 100 μl of HRP-goat-anti-mouse IgG (H+L) (purchased from Beyotime biotechnology research institute, diluted to 1 in 1000 by PBST) was added as the second antibody for another 1 hr incubation. 100 μl of TMB substrate solution (purchased from Beyotime biotechnology research institute) was added after plate had been washed with PBST. The reaction of each well of microplates was terminated by adding 50 μl of 2 M H_2_SO_4_ 30 min. The values of absorbance were measured at the wavelength of 450 nm, and the reference wavelength was 570 nm. Each experiment was repeated more than twice. All steps were done at room temperature.

### Clone and analysis of HPV16 E6, E7 RNA and HPV18 E6, E7 RNA

Genomic DNA was extracted from HPV16 and HPV18 positive samples (gained from Maternal and Child Care Service Centre, Nanjing, China) by using Beyotime genomic DNA extraction kit (Centrifugal column type, Product ID: D0063). The HPV16 E6, E7 and HPV18 E6, E7 genes were amplified by PCR (Length sizes of HPV16 E6 and E7 were 477 bp and 296 bp, length sizes of HPV18 E6 and E7 were 477 bp and 318 bp, respectively) by using primers which containing T7 RNA polyase promoter (underlined). Sequence (5′-3′) of HPV16 E6 ORF sense primer: TAATACGACTCACTATAGGGATGCACCAAAAGAGAACTGCAATGTTTCAG, sequence (5′-3′) of HPV16 E6 ORF anti-sense primer: TTACAGCTGGGTTTCTCTACGTGTTCTTG; sequence (5′-3′) of HPV16 E7 ORF sense primer: TAATACGACTCACTATAGGGATGCATGGAGATACACCTACATTGCAT, sequence (5′-3′) of HPV16 E7 ORF anti-sense primer: TTATGGTTTCTGAGAACAGATGGGGCACAC. Sequence (5′-3′) of HPV18 E6 ORF sense primer: TAATACGACTCACTATAGGGATGGCGCGCTTTGAGGATC, sequence (5′-3′) of HPV18 E6 ORF anti-sense primer: TTATACTTGTGTTTCTCTGCGTCGTTGGAG; sequence (5′-3′) of HPV18 E7 ORF sense primer: TAATACGACTCACTATAGGGATGYATGGACCTAAGGCAACATTGC, sequence (5′-3′) of HPV18 E7 ORF anti-sense primer: TTACTGCTGGGATGCACACCACGGACAC.

The PCR products of HPV16 E6, E7 and HPV18 E6, E7 gene fragments were purfied by gel extraction kit (TIANGEN Universal DNA Purification Kit, Cat.#DP214-02). The pure PCR products were connected to pMD-19T Vector (TaKaRa), and then the recombinant plasmids were transformed into DH5α competent cells. The positive single colony was shaking cultured in LB fluid medium overnight. The recombinant plasmids were then extracted by plasmid extraction kit (TIANpure Midi Plasmid Kit, Cat#DP107-02) and sequenced by GenScript company. The sequencing results were analyzed by blast in NCBI GenBank.

### Preparation of antigen samples of HPV16 E6, E7 and HPV18 E6, E7 RNA

The PCR products of fragments which concentrations were 0.2 μg/μl were transcribed by *in vitro* transcription kit (TaKaRa, D6140). The HPV16 E6, E7 and HPV18 E6, E7 RNA transcribed *in vitro* were check out by 1% AGE (agarose gel electrophoresis) at 110 V for 30 min. The products were then treated with 20 U RNase free DNase I to eliminate DNA template at 37 °C for 30 min, and to inactivate DNase I at 65 °C for 10 min.

The DNA probes which complemented to the HPV16 E6 and E7 RNA were designed according to NCBI published HPV16 E6 and E7 gene sequence and shown in Table 2. The DNA probes hybridized with the gradient dilution of HPV16 E6 and E7 RNAs which were transcribed *in vitro* as the mentioned hybridization system and condition.

### Immunoassay of HPV16 E6 and E7 RNA and the specificity studies

The procedure was same as the immunoassay for detecting *in vitro* transcription products of HPV 16 E6/E7, HPV18 E6/E7 and HepG2 total RNA as described above (the HepG2 cell line was purchased from the cell bank, Chinese academy of sciences). Each experiment was repeated more than twice.

### Immunoassay for detecting HPV+ cell lines and clinic samples

The total RNA of the CaSki and SiHa cell lines (purchased from the cell bank, Chinese academy of sciences as the HPV16+ cells), the clinic sample of HPV16+ oropharyngeal carcinoma (gained from Institute and Hospital of Stomatology, Nanjing, China), and the clinic sample of HPV16+ cervical cancer (gained from Maternal and Child Care Service Centre, Nanjing, China) was extracted by Qiagen RNeasy® Mini Kit (74104) according to the instructions. The total RNA was hybridized with the mixture of HPV16 E6 and E7 DNA probes following the mentioned hybridization system and condition.

## Additional Information

**How to cite this article**: Ding, S. *et al*. Establishment of immunoassay for detecting HPV16 E6 and E7 RNA. *Sci. Rep*. **5**, 13686; doi: 10.1038/srep13686 (2015).

## Figures and Tables

**Table 1:** Synthetic DNA and RNA probes to form hybrids.

Names	Sequences (5′-3′)	Length
HPV16E6DNA1	AGTTACTGCGACGTGAGGTATATGACTTTGCTTTTCGGGA	40 nt
HPV16E6RNA1	UCCCGAAAAGCAAAGUCAUAUACCUCACGUCGCAGUAACU	40 nt
HPV16E6DNA2	CCCGAAAAGCAAAGTCATATACCTCACGTCGCAGTA	36 nt
HPV16E6RNA2	UACUGCGACGUGAGGUAUAUGACUUUGCUUUUCGGG	36 nt
HPV16E7DNA1	GCAAGTGTGACTCTACGCTTCGGTTGTGCGTA	32 nt
HPV16E7RNA1	UACGCACAACCGAAGCGUAGAGUCACACUUGC	32 nt
HPV16E7DNA2	GACAAGCAGAACCGGACAGAGCCCATTACA	30 nt
HPV16E7RNA2	UGUAAUGGGCUCUGUCCGGUUCUGCUUGUC	30 nt

**Table 2:** Probe sequences to detect HPV16 E6 and E7 RNA.

Names	Sequences (5′-3′)	Location
E6 DNA Probe 1	GCTCTGTGCATAACTGTGGTAACTTTCTGGGTCG	43–76
E6 DNA Probe 2	TCCCGAAAAGCAAAGTCATATACCTCACGTCGCAGTAACT	128–167
E6 DNA Probe 3	CAGCTGGGTTTCTCTACGTGTTCTTGATGATCTGCAACAA	435–474
E7 DNA Probe 1	TACGCACAACCGAAGCGTAGAGTCACACTTGC	176–207
E7 DNA Probe 2	AGTGTGCCCATTAACAGGTCTTCCAAAGTACGAATGTCTACGTGTGTGC	212–260
